# Autophagy Regulates the Survival of Hair Cells and Spiral Ganglion Neurons in Cases of Noise, Ototoxic Drug, and Age-Induced Sensorineural Hearing Loss

**DOI:** 10.3389/fncel.2021.760422

**Published:** 2021-10-13

**Authors:** Lingna Guo, Wei Cao, Yuguang Niu, Shuangba He, Renjie Chai, Jianming Yang

**Affiliations:** ^1^State Key Laboratory of Bioelectronics, School of Life Sciences and Technology, Jiangsu Province High-Tech Key Laboratory for Bio-Medical Research, Southeast University, Nanjing, China; ^2^Department of Otolaryngology Head and Neck Surgery, The Second Affiliated Hospital of Anhui Medical University, Hefei, China; ^3^Department of Ambulatory Medicine, The First Medical Center of PLA General Hospital, Beijing, China; ^4^Department of Otolaryngology Head and Neck Surgery, Nanjing Tongren Hospital, School of Medicine, Southeast University, Nanjing, China; ^5^Co-Innovation Center of Neuroregeneration, Nantong University, Nantong, China; ^6^Institute for Stem Cell and Regeneration, Chinese Academy of Sciences, Beijing, China; ^7^Beijing Key Laboratory of Neural Regeneration and Repair, Capital Medical University, Beijing, China

**Keywords:** hearing loss, hair cells, spiral ganglion neurons, autophagy, mechanism

## Abstract

Inner ear hair cells (HCs) and spiral ganglion neurons (SGNs) are the core components of the auditory system. However, they are vulnerable to genetic defects, noise exposure, ototoxic drugs and aging, and loss or damage of HCs and SGNs results in permanent hearing loss due to their limited capacity for spontaneous regeneration in mammals. Many efforts have been made to combat hearing loss including cochlear implants, HC regeneration, gene therapy, and antioxidant drugs. Here we review the role of autophagy in sensorineural hearing loss and the potential targets related to autophagy for the treatment of hearing loss.

## Introduction

According to the World Health Organization (WHO, 2021), about 5% of the world’s population (or 430 million people) suffer from hearing impairment, and it is expected that the number of people with disabling hearing loss will be around 700 million by 2050. Hearing loss is not only a physical and financial burden in social life, but also causes psychological problems and psychiatric disorders, including cognitive decline and depression (Strawbridge et al., 2000; Steffens et al., 2006; Lin et al., 2013). Indeed, hearing loss has become a serious threat to global population health and economic development.

Genetic alterations, noise, ototoxic drugs, and aging can all contribute to hearing loss. Although the causes vary, the most common causes of deafness are damage or loss of hair cells (HCs) and degeneration of spiral ganglion neurons (SGNs). HCs are responsible for converting external sound signals into electrical signals that are transmitted to the brainstem through SGNs (Groves and Fekete, 2012). Recent studies have shown that these sensory cells cannot spontaneously regenerate in adult mammals (Stone et al., 1998; Brigande and Heller, 2009; Cox et al., 2014), so damage or loss of HCs and degeneration of SGNs can result in permanent deafness.

Cochlear implants offer strategies to mitigate hearing loss, but their effectiveness has been reported to be highly correlated with the remaining HCs and SGNs in the cochlea. Efforts have been made to protect HCs and SGNs against noise or ototoxic drugs-induced death, and N-acetylcysteine and neurotrophins have been shown to prevent HC death and SGN degeneration to some extent (Aladag et al., 2016; Chen et al., 2018; Wu et al., 2020). Recently, autophagy has been reported to play an antioxidative role in preventing sensorineural hearing loss (SNHL) (Ye et al., 2019a). In this review, we present the role of autophagy in hearing loss induced by noise exposure, ototoxic drugs and aging, and describe the molecules and signaling pathways involved in autophagy in the inner ear.

## The Mechanism and Process of Autophagy

Autophagy is a highly conserved degradation system in eukaryotic cells that maintains cellular homeostasis, and autophagy can be induced by nutrient deficiency and reactive oxygen species (ROS) accumulation (Mizushima, 2007; Eskelinen, 2019). Through the autophagy pathway, damaged cytoplasmic components are absorbed and transferred to lysosomes, where they are degraded and recycled. There are three main types of autophagy, the most common form being macroautophagy, which is the form generally being referred to by the term “autophagy.” In this process, bilayer organelles called autophagosomes carry cytoplasmic products to lysosomes for degradation (Mizushima, 2007; Mizushima and Komatsu, 2011). This dynamic process generally comprises the following four steps: first is the initiation of autophagy through the envelopment of the cytosolic contents within phagophores; second is the formation of the autophagosome, which is a double-membrane vesicle; third is the fusion of autophagosomes with lysosomes to form autolysosomes; and fourth is the degradation of the contents of the autolysosomes (Feng et al., 2014). The second form is microautophagy, in which the cytoplasmic contents enter the lysosome through direct invagination or through deformation of the lysosomal membrane (Li et al., 2012). The third form is molecular chaperone-mediated autophagy, which is a highly specific process in which proteins containing a KFERQ motif are recognized and transported to the lysosomal membrane (Kaushik and Cuervo, 2018; Yang et al., 2019).

The biogenesis of autophagy requires many autophagy-related (ATG) proteins. So far more than 30 ATGs have been shown to be involved in the initiation and maturation of autophagy (Klionsky et al., 2003; Xie and Klionsky, 2007; Mizushima et al., 2011; Wesselborg and Stork, 2015), and the ATGs that are required for autophagosome formation are divided into several functional units. The autophagy-related 2 (ATG1)–Unc51-like kinase (ULK) complex (ULK1) plays a vital role during the initiation stage, and because this complex is negatively regulated by mammalian target of rapamycin complex 1 (mTORC1) (Noda and Ohsumi, 1998; Kamada et al., 2010), the inactivation of mTORC1 by rapamycin stimulates autophagy. Alternatively, the activation of autophagy can also be regulated by AMP-activated protein kinase (AMPK) (Kim et al., 2011; Li and Chen, 2019). The phosphatidylinositol 3-kinase complex (PI3KC), which is activated by ULK1, participates in the formation of autophagic vesicle membranes. ATG9 is the only known transmembrane protein shown to be involved in the delivery of membrane particles to form autophagosomes (Noda et al., 2000; Webber and Tooze, 2010). During the maturation stage, two ubiquitin-like conjugation systems, the ATG5-ATG12 system and the LC3-PE system, play vital roles in the elongation of autophagosomes (Geng and Klionsky, 2008). After the autophagosome is encapsulated, the autophagosome and lysosome fuse to form the autolysosome through the function of proteins such as SNARE (Itakura et al., 2012).

## The Protective Effect of Autophagy Against SNHL

Autophagy is responsible for normal cell survival and homeostasis. A variety of human conditions, such as neurodegenerative diseases, cancer, and inflammation, have been reported to be associated with dysregulated autophagic processes (Levine et al., 2011; White, 2012; Kochergin and Zakharova, 2016). In the inner ear, many studies have shown that autophagy played an important role in cell development, differentiation, and survival (Fujimoto et al., 2017; Magarinos et al., 2017), and recently there has been renewed interest in regulating autophagy to prevent SNHL.

Noise and ototoxic drugs increased the levels of oxidative stress in HCs, which contributed to cell death (Warchol, 2010; Tabuchi et al., 2011; Sheth et al., 2017; Wu et al., 2020), and in a mouse model that was exposed to noise, the level of autophagy was increased in HCs (Xu et al., 2021). It is worth noting that the oxidative stress level in response to noise was dose dependent, and moderate noise induced temporary threshold shifts and increased the level of autophagy in outer hair cells, while severe noise produced excess ROS that induced permanent threshold shifts (Yuan et al., 2015). Increasing autophagy with rapamycin can reduce the accumulation of ROS and prevent cell death from noise exposure. In contrast, blocking autophagy through the autophagy inhibitor 3-methyladenine (3-MA) or knocking down LC3 can increase the accumulation of ROS and promote cell death (Yuan et al., 2015). More recently, a study reported that treatment with FK506 (tacrolimus), a calcineurin inhibitor, increased autophagy and inhibited ROS and alleviated moderate noise-induced HC damage and hearing loss (He et al., 2021b).

Ototoxic drugs such as aminoglycoside antibiotics and cisplatin can also result in HC damage and hearing loss. He et al. (2017) found that autophagy activity was increased in neomycin or gentamicin-treated HCs and HEI-OC1 cells. Treatment with rapamycin increased autophagy activity and decreased ROS accumulation and apoptosis, while treatment with 3-MA or knockdown of ATG5 resulted in reduced autophagy activity and increased ROS levels and apoptosis. Other studies also showed that upregulation of autophagy alleviated cisplatin-induced ototoxicity in HCs (Fang and Xiao, 2014; Liu et al., 2019;Liang et al., 2021).

Presbycusis (age-related hearing loss) is a common sensory disorder associated with aging. The level of autophagy decreased with age, and the upregulation of autophagy can promote aging HC survival and slow the degeneration of auditory cells (Yuan et al., 2018; He et al., 2021a).

Autophagy also exerts a protective effect in SGNs against ototoxic drug-induced damage. Administration of kanamycin and furosemide induced HC loss and subsequent SGN degeneration by impairing autophagic flux and lysosomal biogenesis, and restoration of autophagy by promoting transcription factor EB (TFEB) translocation into the nucleus attenuated SGN degeneration (Ye et al., 2019b). In cisplatin-induced SGN damage, activation of autophagy by rapamycin alleviated SGN apoptosis and hearing loss, and inhibition of autophagy by 3-MA aggravated the degeneration of SGNs (Liu et al., 2021). Thus, autophagy has a protective effect against HC loss, SGN degeneration and subsequent hearing impairment.

## The Pro-Apoptotic Effect of Autophagy in SNHL

Autophagy has a dual function of pro-survival and pro-apoptotic, which has been demonstrated in many diseases, especially cancers, and the role of autophagy depends on the developmental stage and tumor type (Singh et al., 2018). Several reports have demonstrated the pro-apoptotic role of autophagy in SNHL. In a model of cisplatin-induced HC damage, exposure to 15 μM cisplatin for 48 h induced excessive autophagy, while co-treatment of cisplatin with meclofenamic acid, a highly selective inhibitor of fatmass and obesity-associated enzyme, inhibited the cisplatin-induced excessive autophagy in HEI-OC1 cells and reduced oxidative stress and cell apoptosis (Li et al., 2018). Another study indicated that pretreatment with U0126, an inhibitor of the ERK1/2 signaling pathway, can reduce the level of cisplatin-induced autophagy in HEI-OC1 cells and HCs and can reduce cisplatin-induced ROS and apoptosis (Wang et al., 2021). Interestingly, a study showed that in cisplatin-treated HEI-OC1 cells, autophagy promoted cell survival in the early phase (during the first 8 h) of cisplatin treatment, while autophagy induced cell death in the late phase (Youn et al., 2015).

## Mitophagy in SNHL

Autophagy is considered to be a non-selective process in the degradation of a large number of cytoplasmic components. However, recent studies have shown that there are many types of selective autophagy. Some types of selective autophagy have recently been found in the inner ear, for example, mitophagy and pexophagy. Defective, excessive, and aged mitochondria produce toxic byproducts, particularly ROS, and mitophagy is a specific autophagic process that selectively removes these redundant or damaged mitochondria in order to reduce ROS levels and to maintain the normal function of the mitochondria (Kroemer et al., 2007; Novak, 2012). Mitophagy has been linked to neurodegenerative diseases, cancer, and aging (Bernardini et al., 2017; Chu, 2019; Tran and Reddy, 2021). Recent studies have indicated potential associations between mitophagy and age-related hearing loss, and in the cochlea of aged mice, mitophagy was reduced along with decreased expression of mitophagy-related genes and proteins (Oh et al., 2020; Youn et al., 2020). Damaged mitochondria were increased in HCs and SGNs in aged mice, and activation of mitophagy alleviated cellular senescence by promoting mitochondrial protein degradation (Kim et al., 2021). The same phenomenon was observed in carbonyl cyanide m-chlorophenyl hydrazone-induced cytotoxicity in HEI-OC1 cells and in the organ of Corti, and the protein level of mitochondrial cytochrome c oxidase subunit 4 was downregulated (Setz et al., 2018). However, in aminoglycoside-induced HC loss, neither neomycin nor gentamicin exposure had an impact on the level of mitophagy, thus suggesting a mitophagy-independent pathway of aminoglycoside ototoxicity (He et al., 2017; Setz et al., 2018).

## Pexophagy in SNHL

Pexophagy is another selective autophagy pathway, in which peroxisomes are selectively degraded in vacuoles in response to environmental stimuli (Farre et al., 2009; Germain and Kim, 2020). It has been reported that pexophagy was related to inflammation induced by lipopolysaccharide exposure, and impaired pexophagy resulted in the accumulation of impaired peroxisomes and redox disequilibrium (Vasko et al., 2013). Pexophagy was associated with noise-induced HC damage, overexposure to noise led to an increased level of peroxisome in HCs and SGNs, and defective pexophagy led to noise-induced hearing loss (Delmaghani et al., 2015; Defourny et al., 2019). Pejvakin was a peroxisome-associated protein that directly recruited LC3B to promote pexophagy in order to protect cochlear HCs against noise-induced damage (Defourny et al., 2019).

## Proteins That Modulate Autophagy in SNHL

A number of molecules have been reported to respond to cell damage by regulating autophagy. TFEB is a major regulator of autophagy and lysosomal biogenesis, and phosphorylated TFEB is inactive and remains in the cytoplasm, while dephosphorylated TFEB is translocated to the nucleus where it promotes the transcription of its target genes (Martina et al., 2012; Settembre et al., 2012). In kanamycin-induced degenerated SGNs, TFEB remained in the cytoplasm and the autophagic flux was impaired, while the mTOR inhibitor temsirolimus (CCI-779) promoted the translocation of TFEB to the nucleus thus restoring autophagic flux and ameliorating SGN degeneration (Ye et al., 2019b). Phosphatase and tensin homolog (PTEN)-induced putative kinase 1 (PINK1) also shown a protective effect against gentamicin and cisplatin-induced ototoxicity. PINK1 promoted autophagy and inhibited the P53 pathway in gentamicin-induced HC damage (Yang et al., 2018b), while in response to cisplatin-induced HC and SGN damage, PINK1 induced autophagy and inhibited the JNK signaling pathway (Yang et al., 2018a). Peroxiredoxin 1 (PRDX1) also played a protective role in cisplatin-induced SGN damage by activating autophagy through the activation of the PTEN-AKT signaling pathway (Liu et al., 2021). FoxG1 protected HC and delayed age-related hearing loss via autophagy making it be used as a strategy to delay age-related hearing loss (He et al., 2021a).

In addition, some proteins have detrimental effects regarding ototoxicity. For example, STAT1 is a regulator of cell death and has been reported to participate in cisplatin-induced HC damage. Knockdown of STAT1 by siRNA reduced cisplatin-induced ototoxicity (Kaur et al., 2011), and Levano et al. found that STAT1 played a role in modulating the autophagy pathway, with higher levels of autophagy seen in STAT1−/− explants in response to gentamicin and cisplatin (Levano and Bodmer, 2015). Thus, different molecules and pathways regulate the occurrence and development of SNHL in different contexts.

## miRNAs Related to Autophagy in SNHL

**FIGURE 1:**
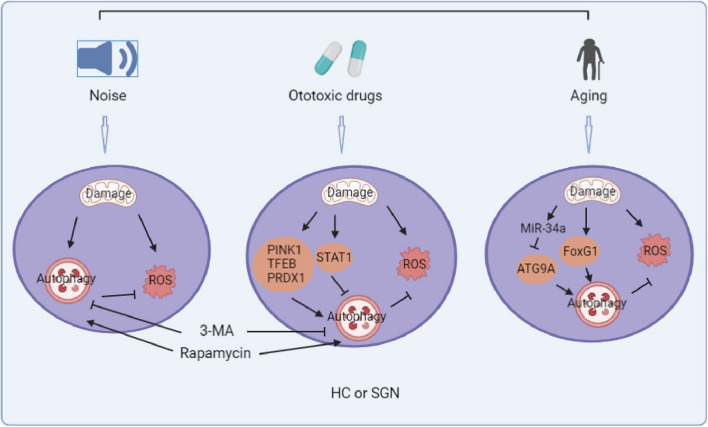
Summary diagram of the role of autophagy in noise, ototoxic drug, and age-induced sensorineural hearing loss. (The picture is created in BioRender.com).

miRNAs are a class of small endogenous RNAs with a length of about 21–23 nucleotides, and they play a variety of important regulatory roles in cells (Rupaimoole and Slack, 2017). miRNAs are potential therapeutic targets for treating cancer and other diseases, and miRNAs are also involved in SNHL (Chen et al., 2019). miR-34a was shown to be associated with age-related hearing loss in mice and humans (Pang et al., 2016, 2017), and miR-34a was activated with aging and overexpression of miR-34a significantly decreased the level of ATG9A thus inhibiting autophagic flux and inducing cell death (Pang et al., 2017). A considerable number of miRNAs have been found to be involved in autophagy cascades, such as miR-204, miR-216a, and miR-375 etc. (Su et al., 2015), but the roles of these miRNAs are poorly studied in relation to SNHL (Figure 1).

## Conclusion

There is no doubt that autophagy plays an important role in SNHL. Although excessive autophagy can lead to cell death under some conditions, activation of autophagy protects HCs and SGNs against oxidative stress-induced death. It is important to be clear that the mechanisms of autophagy are complex and that different stimuli may lead to activation of different pathways. Though we have known that some proteins and miRNAs participate in the autophagic pathways involved in SNHL making them potential targets for treatment of SNHL, however, the specific signaling pathways they participate in remain unclear, let alone the known connections between these proteins and miRNAs. Furthermore, there are potential proteins and miRNAs whose functions in SNHL have not yet been identified. Future studies should thus further clarify the mechanism of autophagy in response to different stimuli in order to develop ways to regulate autophagy and thus protect HCs and SGNs. However, the application of autophagy as a treatment for deafness is still a long way off. Current research has been limited to cell lines, explants and animals, and few clinical trials have examined the role of autophagy. Given the complexity of mechanisms and functions of autophagy, the safest and most effective strategies must be studied in future research.

## Author Contributions

All authors listed have made a substantial, direct and intellectual contribution to the work, and approved it for publication.

## Conflict of Interest

The authors declare that the research was conducted in the absence of any commercial or financial relationships that could be construed as a potential conflict of interest.

## Publisher’s Note

All claims expressed in this article are solely those of the authors and do not necessarily represent those of their affiliated organizations, or those of the publisher, the editors and the reviewers. Any product that may be evaluated in this article, or claim that may be made by its manufacturer, is not guaranteed or endorsed by the publisher.

## References

[B1] Aladag I. Guven M. Songu M. (2016). Prevention of gentamicin ototoxicity with N-acetylcysteine and vitamin A. *J. Laryngol. Otol.* 130 440–446. 10.1017/S0022215116000992 27095551

[B2] Bernardini P. Lazarou M. Dewson G. (2017). Parkin and mitophagy in cancer. *Oncogene* 36 1315–1327. 10.1038/onc.2016.302 27593930

[B3] Brigande J. V. Heller S. (2009). Quo vadis, hair cell regeneration? *Nat. Neurosci.* 12 679–685. 10.1038/nn.2311 19471265 PMC2875075

[B4] Chen H. R. Wijesinghe P. Nunez D. A. (2019). MicroRNAs in acquired sensorineural hearing loss. *J. Laryngol. Otol.* 133 650–657. 10.1017/S0022215119001439 31358070

[B5] Chen Y. Xing L. Xia Chen Z. Yin S. Wang J. (2018). AAV-mediated NT-3 overexpression protects cochleae against noise-induced synaptopathy. *Gene Ther.* 25 251–259. 10.1038/s41434-018-0012-0 29535374 PMC6062503

[B6] Chu C. T. (2019). Mechanisms of selective autophagy and mitophagy: implications for neurodegenerative diseases. *Neurobiol. Dis.* 122 23–34. 10.1016/j.nbd.2018.07.015 30030024 PMC6396690

[B7] Cox B. C. Chai R. J. Lenoir A. Liu Z. Y. Zhang L. L. Nguyen D. H. (2014). Spontaneous hair cell regeneration in the neonatal mouse cochlea in vivo. *Development* 141 816–829. 10.1242/dev.109421 24496619 PMC3912828

[B8] Defourny J. Aghaie A. Perfettini I. Avan P. Delmaghani S. Petit C. (2019). Pejvakin-mediated pexophagy protects auditory hair cells against noise-induced damage. *Proc. Natl. Acad. Sci. U.S.A.* 116 8010–8017. 10.1073/pnas.1821844116 30936319 PMC6475433

[B9] Delmaghani S. Defourny J. Aghaie A. Beurg M. Dulon D. Thelen N. (2015). Hypervulnerability to sound exposure through impaired adaptive proliferation of peroxisomes. *Cell* 163 894–906. 10.1016/j.cell.2015.10.023 26544938

[B10] Eskelinen E. L. (2019). Autophagy: supporting cellular and organismal homeostasis by self-eating. *Int. J. Biochem. Cell Biol.* 111 1–10. 10.1016/j.biocel.2019.03.010 30940605

[B11] Fang B. Xiao H. J. (2014). Rapamycin alleviates cisplatin-induced ototoxicity in vivo. *Biochem. Biophys. Res. Commun.* 448 443–447. 10.1016/j.bbrc.2014.04.123 24796670

[B12] Farre J. C. Krick R. Subramani S. Thumm M. (2009). Turnover of organelles by autophagy in yeast. *Curr. Opin. Cell Biol.* 21 522–530. 10.1016/j.ceb.2009.04.015 19515549 PMC2725217

[B13] Feng Y. C. He D. Yao Z. Y. Klionsky D. J. (2014). The machinery of macroautophagy. *Cell Res.* 24 24–41. 10.1038/cr.2013.168 24366339 PMC3879710

[B14] Fujimoto C. Iwasaki S. Urata S. Morishita H. Sakamaki Y. Fujioka M. (2017). Autophagy is essential for hearing in mice. *Cell Death Dis.* 8:e2780. 10.1038/cddis.2017.194 28492547 PMC5520715

[B15] Geng J. Klionsky D. J. (2008). The Atg8 and Atg12 ubiquitin-like conjugation systems in macroautophagy. ‘Protein modifications: beyond the usual suspects’ review series. *EMBO Rep.* 9 859–864. 10.1038/embor.2008.163 18704115 PMC2529362

[B16] Germain K. Kim P. K. (2020). Pexophagy: a model for selective autophagy. *Int. J. Mol. Sci.* 21:578. 10.3390/ijms21020578 PMC7013971 31963200

[B17] Groves K. Fekete D. M. (2012). Shaping sound in space: the regulation of inner ear patterning. *Development* 139 245–257. 10.1242/dev.078257 22186725 PMC3243092

[B18] He Z. H. Guo L. N. Shu Y. L. Fang Q. J. Zhou H. Liu Y. Z. (2017). Autophagy protects auditory hair cells against neomycin-induced damage. *Autophagy* 13 1884–1904. 10.1080/15548627.2017.1359449 28968134 PMC5788479

[B19] He Z. H. Pan S. Zheng H. W. Fang Q. J. Hill K. Sha S. H. (2021b). Treatment with calcineurin inhibitor FK506 attenuates noise-induced hearing loss. *Front. Cell Dev. Biol.* 9:648461. 10.3389/fcell.2021.648461 33777956 PMC7994600

[B20] He Z. H. Li M. Fang Q. J. Liao F. L. Zou S. Y. Wu X. (2021a). FOXG1 promotes aging inner ear hair cell survival through activation of the autophagy pathway. *Autophagy.* 10.1080/15548627.2021.1916194 [Epub ahead of print]. 34006186 PMC8726647

[B21] Itakura E. Kishi-Itakura C. Mizushima N. (2012). The hairpin-type tail-anchored SNARE syntaxin 17 targets to autophagosomes for fusion with endosomes/lysosomes. *Cell* 151 1256–1269. 10.1016/j.cell.2012.11.001 23217709

[B22] Kamada Y. Yoshino K. Kondo C. Kawamata T. Oshiro N. Yonezawa K. (2010). Tor directly controls the Atg1 kinase complex to regulate autophagy. *Mol. Cell. Biol.* 30 1049–1058. 10.1128/Mcb.01344-09 19995911 PMC2815578

[B23] Kaur T. Mukherjea D. Sheehan K. Jajoo S. Rybak L. P. Ramkumar V. (2011). Short interfering RNA against STAT1 attenuates cisplatin-induced ototoxicity in the rat by suppressing inflammation. *Cell Death Dis.* 2:e180. 21776018 10.1038/cddis.2011.63 PMC3199718

[B24] Kaushik S. Cuervo A. M. (2018). The coming of age of chaperone-mediated autophagy. *Nat. Rev. Mol. Cell Biol.* 19 365–381. 10.1038/s41580-018-0001-6 29626215 PMC6399518

[B25] Kim J. Kundu M. Viollet B. Guan K. L. (2011). AMPK and mTOR regulate autophagy through direct phosphorylation of Ulk1. *Nat. Cell Biol.* 13 132–141. 10.1038/ncb2152 21258367 PMC3987946

[B26] Kim Y. J. Choo O. S. Lee J. S. Jang J. H. Woo H. G. Choung Y. H. (2021). BCL2 interacting protein 3-like/NIX-mediated mitophagy plays an important role in the process of age-related hearing loss. *Neuroscience* 455 39–51. 10.1016/j.neuroscience.2020.12.005 33346118

[B27] Klionsky D. J. Cregg J. M. Dunn W. A. Emr S. D. Sakai Y.. Sandoval I. V. (2003). A unified nomenclature for yeast autophagy-related genes. *Dev. Cell* 5 539–545. 10.1016/S1534-5807(03)00296-X 14536056

[B28] Kochergin J. A. Zakharova M. N. (2016). The role of autophagy in neurodegenerative diseases. *Neurochem. J.* 10 7–18. 10.1134/S1819712416010098

[B29] Kroemer G. Galluzzi L. Brenner C. (2007). Mitochondrial membrane permeabilization in cell death. *Physiol. Rev.* 87 99–163. 10.1152/physrev.00013.2006 17237344

[B30] Levano S. Bodmer D. (2015). Loss of STAT1 protects hair cells from ototoxicity through modulation of STAT3, c-Jun, Akt, and autophagy factors. *Cell Death Dis.* 6:e2019. 26673664 10.1038/cddis.2015.362 PMC4720895

[B31] Levine B. Mizushima N. Virgin H. W. (2011). Autophagy in immunity and inflammation. *Nature* 469 323–335. 10.1038/nature09782 21248839 PMC3131688

[B32] Li H. Song Y. D. He Z. H. Chen X. Y. Wu X. M. Li X. F. (2018). Meclofenamic acid reduces reactive oxygen species accumulation and apoptosis, inhibits excessive autophagy, and protects hair cell-like HEI-OC1 cells from cisplatin-induced damage. *Front. Cell. Neurosci.* 12:139. 10.3389/fncel.2018.00139 29875633 PMC5974247

[B33] Li J. Chen Y. Y. (2019). AMPK and autophagy. autophagy: biology and diseases. *Basic Sci.* 1206 85–108. 10.1007/978-981-15-0602-4_4 31776981

[B34] Li W. W. Li J. Bao J. K. (2012). Microautophagy: lesser-known self-eating. *Cell. Mol. Life Sci.* 69 1125–1136. 10.1007/s00018-011-0865-5 22080117 PMC11114512

[B35] Liang Z. R. Zhang T. Zhan T. Cheng G. Zhang W. J. Jia H. Y. (2021). Metformin alleviates cisplatin-induced ototoxicity by autophagy induction possibly via the AMPK/FOXO3a pathway. *J. Neurophysiol.* 125 1202–1212. 10.1152/jn.00417.2020 33625942

[B36] Lin F. R. Yaffe K. Xia J. Xue Q. L. Harris T. B. Purchase-Helzner E. (2013). Hearing loss and cognitive decline in older adults. *JAMA Int. Med.* 173 293–299. 10.1001/jamainternmed.2013.1868 PMC3869227 23337978

[B37] Liu T. Y. Zong S. M. Luo P. Qu Y. J. Wen Y. Y. Du P. Y. (2019). Enhancing autophagy by down-regulating GSK-3 beta alleviates cisplatin-induced ototoxicity in vivo and in vitro. *Toxicol. Lett.* 313 11–18. 10.1016/j.toxlet.2019.05.025 31220555

[B38] Liu W. W. Xu L. Wang X. Zhang D. G. Sun G. Y. Wang M. (2021). PRDX1 activates autophagy via the PTEN-AKT signaling pathway to protect against cisplatin-induced spiral ganglion neuron damage. *Autophagy.* 10.1080/15548627.2021.1905466 [Epub ahead of print]. 33749526 PMC8726717

[B39] Magarinos M. Pulido S. Aburto M. R. Rodriguez R. D. Varela-Nieto I. (2017). Autophagy in the vertebrate inner ear. *Front. Cell Dev. Biol.* 5:56. 10.3389/fcell.2017.00056 28603711 PMC5445191

[B40] Martina J. A. Chen Y. Gucek M. Puertollano R. (2012). MTORC1 functions as a transcriptional regulator of autophagy by preventing nuclear transport of TFEB. *Autophagy* 8 903–914. 10.4161/auto.19653 22576015 PMC3427256

[B41] Mizushima N. (2007). Autophagy: process and function. *Genes Dev.* 21 2861–2873. 10.1101/gad.1599207 18006683

[B42] Mizushima N. Komatsu M. (2011). Autophagy: renovation of cells and tissues. *Cell* 147 728–741. 10.1016/j.cell.2011.10.026 22078875

[B43] Mizushima N. Yoshimori T. Ohsumi Y. (2011). The role of Atg proteins in autophagosome formation. *Ann. Rev. Cell Dev. Biol.* 27 107–132. 10.1146/annurev-cellbio-092910-154005 21801009

[B44] Noda T. Ohsumi Y. (1998). Tor, a phosphatidylinositol kinase homologue, controls autophagy in yeast. *J. Biol. Chem.* 273 3963–3966. 10.1074/jbc.273.7.3963 9461583

[B45] Noda T. Kim J. Huang W. P. Baba M. Tokunaga C. Ohsumi Y. (2000). Apg9p/Cvt7p is an integral membrane protein required for transport vesicle formation in the Cvt and autophagy pathways. *J. Cell Biol.* 148 465–479. 10.1083/jcb.148.3.465 10662773 PMC2174799

[B46] Novak I. (2012). Mitophagy: a complex mechanism of mitochondrial removal. *Antioxid. Redox Signal.* 17 794–802. 10.1089/ars.2011.4407 22077334

[B47] Oh J. Youn C. K. Jun Y. Jo E. R. Cho S. I. (2020). Reduced mitophagy in the cochlea of aged C57BL/6J mice. *Exp. Gerontol.* 137:110946. 10.1016/j.exger.2020.110946 32387126

[B48] Pang J. Q. Xiong H. Lin P. L. Lai L. Yang H. D. Liu Y. M. (2017). Activation of miR-34a impairs autophagic flux and promotes cochlear cell death via repressing ATG9A: implications for age-related hearing loss. *Cell Death Dis.* 8:e3079. 28981097 10.1038/cddis.2017.462 PMC5680584

[B49] Pang J. Q. Xiong H. Yang H. D. Ou Y. K. Xu Y. D. Huang Q. H. (2016). Circulating miR-34a levels correlate with age-related hearing loss in mice and humans. *Exp. Gerontol.* 76 58–67. 10.1016/j.exger.2016.01.009 26802970

[B50] Rupaimoole R. Slack F. J. (2017). MicroRNA therapeutics: towards a new era for the management of cancer and other diseases. *Nat. Rev. Drug Discov.* 16 203–221. 10.1038/nrd.2016.246 28209991

[B51] Settembre C. Zoncu R. Medina D. L. Vetrini F. Erdin S. Erdin S. (2012). A lysosome-to-nucleus signalling mechanism senses and regulates the lysosome via mTOR and TFEB. *Embo J.* 31 1095–1108. 10.1038/emboj.2012.32 22343943 PMC3298007

[B52] Setz C. Benischke A. S. Bento A. C. P. F. Brand Y. Levano S. Paech F. (2018). Induction of mitophagy in the HEI-OC1 auditory cell line and activation of the Atg12/LC3 pathway in the organ of Corti. *Hear. Res.* 361 52–65. 10.1016/j.heares.2018.01.003 29352609

[B53] Sheth S. Mukherjea D. Rybak L. P. Ramkumar V. (2017). Mechanisms of cisplatin-induced ototoxicity and otoprotection. *Front. Cell. Neurosci.* 11:338. 10.3389/fncel.2017.00338 29163050 PMC5663723

[B54] Singh S. S. Vats S. Chia A. Y. Q. Tan T. Z. Deng S. Ong M. S. (2018). Dual role of autophagy in hallmarks of cancer. *Oncogene* 37 1142–1158. 10.1038/s41388-017-0046-6 29255248

[B55] Steffens D. C. Otey E. Alexopoulos G. S. Butters M. A. Cuthbert B. Ganguli M. (2006). Perspectives on depression, mild cognitive impairment, and cognitive decline. *Arch. Gen. Psychiatry* 63 130–138. 10.1001/archpsyc.63.2.130 16461855

[B56] Stone J. S. Oesterle E. C. Rubel E. W. (1998). Recent insights into regeneration of auditory and vestibular hair cells. *Curr. Opin. Neurol.* 11 17–24. 10.1097/00019052-199802000-00004 9484612

[B57] Strawbridge W. J. Wallhagen M. I. Shema S. J. Kaplan G. A. (2000). Negative consequences of hearing impairment in old age: a longitudinal analysis. *Gerontologist* 40 320–326. 10.1093/geront/40.3.320 10853526

[B58] Su Z. Y. Yang Z. Z. Xu Y. Q. Chen Y. B. Yu Q. (2015). MicroRNAs in apoptosis, autophagy and necroptosis. *Oncotarget* 6 8474–8490. 10.18632/oncotarget.3523 25893379 PMC4496162

[B59] Tabuchi K. Nishimura B. Nakamagoe M. Hayashi K. Nakayama M. Hara A. (2011). Ototoxicity: mechanisms of cochlear impairment and its prevention. *Curr. Med. Chem.* 18 4866–4871. 10.2174/092986711797535254 21919841

[B60] Tran M. Reddy P. H. (2021). Defective autophagy and mitophagy in aging and Alzheimer’s disease. *Front. Neurosci.* 14:612757. 10.3389/fnins.2020.612757 33488352 PMC7820371

[B61] Vasko R. Ratliff B. B. Bohr S. Nadel E. Chen J. Xavier S. (2013). Endothelial peroxisomal dysfunction and impaired pexophagy promotes oxidative damage in lipopolysaccharide-induced acute kidney injury. *Antioxid. Redox Signal.* 19 211–230. 10.1089/ars.2012.4768 23088293 PMC3691927

[B62] Wang D. Shi S. M. Zhang Y. P. Guo P. Wang J. L. Wang W. Q. (2021). U0126 pretreatment inhibits cisplatin-induced apoptosis and autophagy in HEI-OC1 cells and cochlear hair cells. *Toxicol. Appl. Pharmacol.* 415:115447. 33577918 10.1016/j.taap.2021.115447

[B63] Warchol M. E. (2010). Cellular mechanisms of aminoglycoside ototoxicity. *Curr. Opin. Otolaryngol. Head and Neck Surg.* 18 454–458. 10.1097/MOO.0b013e32833e05ec 20717031

[B64] Webber L. Tooze S. A. (2010). New insights into the function of Atg9. *Febs Lett.* 584 1319–1326. 10.1016/j.febslet.2010.01.020 20083107

[B65] Wesselborg S. Stork B. (2015). Autophagy signal transduction by ATG proteins: from hierarchies to networks. *Cell. Mol. Life Sci.* 72 4721–4757. 10.1007/s00018-015-2034-8 26390974 PMC4648967

[B66] White E. (2012). Deconvoluting the context-dependent role for autophagy in cancer. *Nat. Rev. Cancer* 12 401–410. 10.1038/nrc3262 22534666 PMC3664381

[B67] WHO (2021). *Deafness and Hearing Loss.* Available online at: https://www.who.int/news-room/fact-sheets/detail/deafness-and-hearing-loss (accessed April 1, 2021).

[B68] Wu F. Xiong H. Sha S. H. (2020). Noise-induced loss of sensory hair cells is mediated by ROS/AMPK alpha pathway. *Redox Biol.* 29:101406. 31926629 10.1016/j.redox.2019.101406 PMC6933152

[B69] Xie Z. P. Klionsky D. J. (2007). Autophagosome formation: core machinery and adaptations. *Nat. Cell Biol.* 9 1102–1109. 10.1038/ncb1007-1102 17909521

[B70] Xu L. Cheng Y. J. Yan W. Y. (2021). Up-regulation of autophagy and apoptosis of cochlear hair cells in mouse models for deafness. *Arch. Med. Sci.* 17 535–541. 33747288 10.5114/aoms.2018.75348 PMC7959062

[B71] Yang Q. Q. Zhou Y. W. Yin H. Y. Li H. R. Zhou M. J. Sun G. Y. (2018b). PINK1 protects against gentamicin-induced sensory hair cell damage: possible relation to induction of autophagy and inhibition of p53 signal pathway. *Front. Mol. Neurosci.* 11:403. 10.3389/fnmol.2018.00403 30483050 PMC6240688

[B72] Yang Q. Q. Sun G. Y. Yin H. Y. Li H. R. Cao Z. X. Wang J. H. (2018a). PINK1 protects auditory hair cells and spiral ganglion neurons from cisplatin-induced ototoxicity via inducing autophagy and inhibiting JNK signaling pathway. *Free Radic. Biol. Med.* 120 342–355. 10.1016/j.freeradbiomed.2018.02.025 29458150

[B73] Yang R. Wang L. Zhu L. (2019). Chaperone mediated autophagy. autophagy: biology and diseases. *Basic Sci.* 1206 435–452. 10.1007/978-981-15-0602-4_20 31776997

[B74] Ye B. Fan C. Shen Y. L. Wang Q. Hu H. X. Xiang M. L. (2019a). The antioxidative role of autophagy in hearing loss. *Front. Neurosci.* 12:1010. 10.3389/fnins.2018.01010 30686976 PMC6333736

[B75] Ye B. Wang Q. Hu H. X. Shen Y. L. Fan C. Chen P. H. (2019b). Restoring autophagic flux attenuates cochlear spiral ganglion neuron degeneration by promoting TFEB nuclear translocation via inhibiting MTOR. *Autophagy* 15 998–1016. 10.1080/15548627.2019.1569926 30706760 PMC6526833

[B76] Youn C. K. Jun Y. Jo E. R. Cho S. I. (2020). Age related hearing loss in C57BL/6J mice is associated with mitophagy impairment in the central auditory system. *Int. J. Mol. Sci.* 21:7202. 10.3390/ijms21197202 PMC7584026 33003463

[B77] Youn C. K. Kim J. Park J. H. Do N. Y. Cho S. I. (2015). Role of autophagy in cisplatin-induced ototoxicity. *Int. J. Pediatr. Otorhinolaryngol.* 79 1814–1819. 10.1016/j.ijporl.2015.08.012 26307546

[B78] Yuan H. Wang X. R. Hill K. Chen J. Lemasters J. Yang S. M. (2015). Autophagy attenuates noise induced hearing loss by reducing oxidative stress. *Antioxid. Redox Signal.* 22 1308–1324. 10.1089/ars.2014.6004 25694169 PMC4410759

[B79] Yuan J. Zhao X. Y. Hu Y. J. Sun H. Y. Gong G. Q. Huang X. (2018). Autophagy regulates the degeneration of the auditory cortex through the AMPK-mTOR-ULK1 signaling pathway. *Int. J. Mol. Med.* 41 2086–2098. 10.3892/ijmm.2018.3393 29344647 PMC5810242

